# Phytochemical Screening and Multivariate Analysis on Physicochemical and Nutraceutical Value of *Kocho* from False Banana (Enset)

**DOI:** 10.1155/2023/6666635

**Published:** 2023-03-09

**Authors:** Tewodros Birhanu, Tesfaye Adiko, Ramesh Duraisamy

**Affiliations:** Department of Chemistry, College of Natural Sciences, Arba Minch University, SNNPR, Ethiopia

## Abstract

Enset (*Ensete ventricosum*) is one of Ethiopia's most important food crops. The objective of the present study is to evaluate (using multivariate analysis) the effect of fermentation time, varietal differences, and treatment with *gammicho* on the physicochemical and nutraceuticals of *kocho* obtained from false banana in highly cultivated areas such as Disa Kera and Koysha Gorta of Dawro zone, Loma Woreda, South Nations' Nationalities People Regions, Ethiopia. The analyses were carried out for fresh and fermented (with and without local starter, gammicho) enset *kocho* varieties (Meazia and Katania) harvested in two locations. Statistical analysis of the acquired data was performed using Minitab software version 19. It was discovered that each factor influenced significantly (*p* ≤ 0.05) the qualities of kocho independently and with interaction. After four months of fermentation with gammicho, various parameters such as fat (1.69 to 0.62%), fiber (11.46 to 2.79%), pH (6.50 to 3.00), and moisture were dramatically decreased (9.34 to 2.8%). On the other hand, some dietary elements in both kinds were reduced with increasing fermentation time, including ash (2.07 to 3.57%), protein (3.08 to 5.52%), and carbs (71.87 to 84.55%). The results of this study suggest that Meazia has superior physicochemical and nutritional qualities over Katania.

## 1. Introduction

Traditional foods are common diets that address the food and nutrition security of most rural areas in Ethiopia. They are usually obtained from roots and tubers and are higher in quantity per hectare of land than other crops [1]. Enset (*Ensete ventricosum* (Welw.)) is a herbaceous monocot, a large banana-like plant that belongs to the order *Scitamineae*, the family *Musaceae*, and the genus *Ensete* which grows 4-8 meters (sometimes even up to 11 meters) in height. They are perennial monocarpic crops; they produce flowers only once at the end of their maturation and eventually die [2–4]. Enset is the staple crop of sustainable indigenous Ethiopian plants that helps ensure food security in Ethiopia's southern and southwestern highlands. It is similar to the banana plant and cultivated primarily for the large quantity of carbohydrate-rich foods found in a false stem (pseudostem) and an underground corm [5, 6]. Enset has huge biological dynamic components contributing to drug and nutraceutical applications.

Enset has a more prominent measure of starch. Its swelling capacity and solvency values were inferior to potato starch but much more significant than maize starch [7]. Enset starch has momentous drug applications, including tablet binder and disintegrant, super-disintegrants, drug gelling agents, and sustain release agents [8].

The enset plant and its parts add to the native ethnomedicinal values of the general public. Enset is utilized as a staple and costaple nourishment for many Ethiopians. Products from enset are used in various forms in daily and present-day medication, including removing the placenta, relaxing bone break, and antimicrobial activity against viral, bacterial, fungal, and nematode sicknesses of people [8]. Phytochemical examination of enset corms revealed the presence of alkaloids, steroids, phenolics, glycoside, and sugars, segregated from the corm of enset and assessed based on the physical and ghastly information [9]. The Ayurvedic arrangement of medication pseudostem and corm of enset is used to treat different human ailments like diabetes, kidney stone, leucorrhoea, measles, stomachache, antiviral, antifertility, cardiovascular, respiratory, and simple conveyance [10].

Enset is the primary source of traditional fermented starchy foods stored in the trunk's underground stem and lower parts [11]. The corm and pseudostem of the plant are traditionally processed into primary food products such as *kocho*, *bulla*, and *amicho*. *Kocho* is the bulk of fermented starch obtained from the scarped leaf sheaths and the grated corm (underground stem). *Bulla* is a small amount of water-insoluble starchy product made by dehydration of milky juice from *kocho* during processing and allowed to dry. *Amicho* is a boiled enset corm with good flavor and tastes like cooked potato [1]. The nutritional quality of *kocho* is highly affected by processing methods, fermentation time, plant age, varietal differences, place of cultivation, and harvesting time [12]. The length of fermentation varies with the ambient temperature of incubation. In cold temperatures, *kocho* kept more extended fermentation periods (from several months to years) in a pit. Still, fermentation is completed from a week to three months in a warmer area. Even though there is high production of enset plantation, the utilization is insignificant due to a lack of scientific knowledge about the plant and its products [13]. Consumers lack nutritional information and awareness about the variety and fermentation stage at which quality products are obtained. Thus, it is required to assess phytochemicals, the effect of fermentation time, varietal differences, and treatment with *gammicho* on the physicochemical and nutritional compositions of *kocho* obtained from false banana in a highly cultivated area, Dawro, Ethiopia.

Traditionally, people use a starter culture to reduce fermentation time, increase sensory acceptance in different regions, increase its nutritional value, increase its market price, and produce quality products. There is limited research on starter culture technology for Ethiopian traditional fermented *kocho* food in this locality. However, analysis is being carried out to understand the microbial contents and dynamics of gammicho. This study reports its cultural preparation, application, and effect of processing variables on the nutritional and physicochemical properties.

## 2. Materials and Methods

### 2.1. Sources of Sample

The samples (enset, *kocho*, and gammicho) were collected from Dawro zone, Loma Woreda. It is located 500 km from Addis Ababa and 275 km from Hawassa, the capital city of Southern Nation Nationalities and Peoples Regional State, Ethiopia. Two experienced women in *kocho* processing were involved in the selection of enset varieties and their processing of *kocho*. Matured enset plants of the two varieties were selected by the women from their farms and processed traditionally for the desired experiments. The selected two local varieties were identified and authenticated by experts from the Department of Biology at Arba Minch University.

### 2.2. Preparation of Gammicho

In Dawro, the fermenting agent called gammicho (starter culture) was prepared by taking some portion of the false banana plant, which has a pleasing aroma when it started to decay after being removed from the corm, chopped and mixed with herbs (in Amharic: “Kaatia,” “Shuke,” and “Wada Mata”) which have a good flavor, and then crushed together. The mixture is fermented for over two weeks (15 to 20 days) before mixing with *kocho* [14].

### 2.3. Preparation of Enset for Kocho Fermentation

After the complete fermentation of the starter culture (gammicho), the selected enset plants were cut and processed into the fresh dough and apportioned into treatments.

The treatments were as follows: (A) fermentation with a traditional starter and (B) fermenting mass without a starter culture. For treatment (A), the measured amount of fermented starter was chopped and thoroughly mixed with fresh enset dough (in a small bucket), covered with enset leaves, and fermented in a pit. The same experiment was conducted in the two locations with different environmental conditions that kept the other conditions similar.

### 2.4. Collection of Fermented Kocho Sample and Preparation

*Kocho* samples processed from Meazia and Katania varieties fermented at three stages (fresh, two months, and four months) with and without gammicho were collected separately from the selected Kebeles (villages) such as Disa Kera and Koysha Gorta of Dawro zone, Loma Woreda. About 500 g samples of *kocho* from each variety were collected after the bulk product to maintain its homogeneity. They were kept in clean plastic bags that were sealed airtight and transported to the laboratory for further analysis. About 80 g of wet *kocho* samples of each variety at each fermentation stage was taken to a freeze dryer and dried for 36 hours until the constant weight was obtained. The dried samples were ground well in a mortar using a pestle, sieved (320 *μ*m) to remove fibers, kept in unused new glass bottles, and refrigerated for analysis.

### 2.5. Phytochemical Screening

The pilot phytochemical screening of different bioactive compounds (tannins, saponins, alkaloids, polyphenols, terpenoids, flavonoids, and volatile oils) from enset varieties (Meazia, and Katania) are studied as per the customary procedure described earlier [15].

### 2.6. Physicochemical and Nutraceutical Analysis

Moisture, pH, titrable acidity, ash content, fat, fiber, and protein (%*N* × 6.25) were determined according to AOAC standard methods [16]. The pH values of replicate samples were determined using a portable pH meter, calibrated in buffer solutions with pH 4 and 7 [16]. Total carbohydrate was determined by subtracting all nutritional components from hundred according to FAO/WHO, 1998 [17] as

(1) $$\begin{array}{c} \text{Available carbohydrate content }\left(\%\right)=100\%-\left(\%M+\%P+\%F+\%A+\%\text{FI}\right), \end{array}$$

where %*M* is the moisture content, %*P* is the crude protein content, %*F* is the crude fat content, %*A* is the ash content, and %FI is the fiber content.

The gross energy content of a food depends on the components of the food that provide energy (carbohydrates, fat, and protein). It is obtained by multiplying the mean value of crude fat, crude protein, and total carbohydrate by 9, 4, and 4, respectively, as described in literature [17]. It was calculated as

(2) $$\begin{array}{c} \text{Energy }\left(\text{kcal}\right)=\left[\left(4\text{ kcal}./\text{g}\right)\text{g CHO}+\left.\text{g protein}\right)+\left(9\text{ kcal}./\text{g}\right)\text{ g fat}\right]. \end{array}$$

### 2.7. Statistical Analysis

All experiments and analyses were performed in triplicate (data set table) given in supplementary file (available here) of the article; data were expressed as mean ± standard deviation (s.d.). Statistical data analysis was done using Minitab version 19 statistical software. It was used for checking the significant difference among the mean value of nutrient and physicochemical properties of *kocho* against location, fermentation time, variety, and *gammicho* at a 95% confidence interval.

## 3. Results and Discussion

### 3.1. Phytochemical Screening

The results achieved from the phytochemical investigation of enset collected from the different locations (Disa Kera and Koysha Gorta) show encouraging responses in both varieties (Meazia, and Katania) of enset, which contain tannin, saponins, alkaloids, phenols, and flavonoids (as shown in Table 1) that might be available with different amounts. These ingredients were reported to have therapeutic and physiological activities in living beings [18].

The phenolic compounds have biological properties like antiapoptosis, antiaging, anticarcinogen, anti-inflammation, cardiovascular safety, reticence of angiogenesis, and cell multiplication exercises [19]. A few reports have named the antioxidant characteristics of plant products rich in phenolic compounds [20, 21], like flavonoids, phenolic acids, and tocopherols [22]. Tannins tie to proline-rich protein and confine protein synthesis. Flavonoids are hydroxylated phenolic substances perceived to be synthesized by plants in response to microbial disease, and they are antimicrobial substances against a vast gathering of microorganisms in vitro. They likewise are dynamic antioxidants and show anticancer solid activities [23–25]. The studied enset samples additionally contain saponins known to deliver an inhibitory impact on inflammation [26]. Saponins have the property of precipitating and coagulating red platelets. Alkaloids have been associated with therapeutic uses for quite a long time, and one of their common biological properties is their cytotoxicity [27].

### 3.2. Nutraceutical Analysis of Kocho

The experimental results were evaluated statistically, and their corresponding *p* values and other significant ANOVA results are obtained and presented in Table 2. The statistical results showed that the analyzed data are meaningful with lower *p* values (*p* ≤ 0.001) in most cases. Also, the data analysis of all the responses was found to have better significant (*p* ≤ 0.001) interactions among each and solely with a high confidence level based on *R*^2^ and adjusted *R*^2^ (except TA).

The value *R*^2^, which is >95%, indicated strong correlations between experimental results and predicted values. Similarly, lower standard deviations showed relatively accurate empirical data to the actual results. Meanwhile, the adjusted *R*^2^ values of >85% showed satisfactory confirmation of the significance of the model. The model's suitability was studied using the analysis of variance (ANOVA). The overall value for Prob > F which is lower than 0.05 indicated the model terms as significant.

The results in Table 2 show that in the current study, all 4 factors (location, variety, starter, and fermentation time) had a significant impact on the properties of *kocho* food (except in protein, *p* ≥ 0.05). However, the location interacted with other factors, which significantly differed in lower *p* values with 2-way and 3-way interactions (shown in Table 2). Similarly, all the other nutritional food constituents and physicochemical properties (responses) significantly impact the fermentation time, location, variety, and starter addition. Hence, *p* values (the response parameters are categorized as moisture, fat, ash, energy, and pH) had been influenced by all four factors as in 4-way interactions; and other responses such as fiber content, protein, carbohydrates, and TA values have also had significantly influenced as in 3-way and lower factorial interactions. Thus, according to *p* values, the mean values of each respondent's proximate composition and physical properties of *kocho* samples are separated and presented in Tables 3 and 4.

#### 3.2.1. Crude Protein Content

Fermentation time, variation in genetic composition, and treatment with gammicho brought a noticeable change (*p* ≤ 0.05) in the protein content of traditionally fermented enset food (*kocho*). But, the protein content of the samples collected from Disa Kera and Koysha Gorta Kebeles does not show any statistical significance, with a *p* ≥ 0.05 (Table 2), whereas sample location interacts with other factors such as varieties, starter, and fermentation time, which shows *p* ≤ 0.05. It reveals that the model was statistically significant at a 100% confidence level and gives insight into the interaction patterns [28, 29]. Since protein (glycol-protein) is the structural component of living cells, the Meazia variety of *kocho* with a large size contains a slightly higher amount of protein than the Katania variety with a smaller size. The difference in protein contents is due to whether there are protein bodies alongside starch granules in the plant tissue. The cell wall of a plant is made of cellulose by which glycine-rich proteins cross-linked in between different cellulose structures [30]. Also, the protein content of the currently studied samples was higher than the reported *kocho* variety [31], which shows that the presently studied location has a better *kocho* variety (as fresh and fermented) for consumption to enrich the protein.

During fermentation, treating the fresh mass with a traditional starter could accelerate the process by improving the microbial potential of the *kocho*. Meazia and Katania *kocho* fermented with gammicho (V_1_S_1_: 4.49 ± 0.01 and V_2_S_1_: 4.01 ± 0.03) have significantly higher crude protein contents (shown in Table 2) than untreated ones in both studies areas (Disa Kera, DK, and Koysha Gorta, KG Kebeles). This variation could result from the proliferation of microbial cells in a prefermented traditional culture that facilitated enzymatic degradation of polypeptides into smaller peptides, free amino acids, and nonprotein nitrogen that increased the amount of protein in fermented mass.

Fermentation brought a change in the protein content of *kocho.* The results showed (Table 3) that the longer the fermentation time, the increased the protein content of the fermented mash. This increase in the relative amount of protein could be due to the more significant loss of nonprotein than protein components during fermentation (e.g., carbon dioxide) and an underestimation of protein in the starting material because it was tightly associated with starch or cell walls.

The amount of crude protein in *kocho* obtained from the Meazia variety fermented during four months was significantly (*p* < 0.05) increased (3.26 to 5.56% and 3.75 to 5.03%) than that of unfermented mass in both Kebeles. The improvement was found as more in DK than KG (Table 2), which is due to the better performance of microorganisms in hot temperatures which means that the sample site DK is located on low land. Thus, fermenting microorganisms synthesized the structural proteins integral to a microbial cell using free amino acids and nonprotein nitrogen [32]. This was shown from the amino acid profile reported [14] as the fermentation progressed, and some amino acid concentrations may decrease. Further, earlier studies confirmed that the reduction in some amino acid content of pearl millet fermented for 24 hours was used by microorganisms to synthesize a protein that improved the protein content in fermented food products [33, 34].

The highest value (L_1_V_1_F_4_: 5.56%) of crude protein was obtained from *kocho* of Meazia enset variety fermented for four months with gammicho in DK, and the least value was obtained from the untreated fresh mass (see Table 3) of the Katania variety in KG (L_2_V_2_F_0_: 3.08%). A less gradual increment in protein content of *kocho* processed in KG as compared to DK is mainly due to the slow activity of fermenting microorganisms and the inhibition of microbial enzymes by external factors. Since temperature is one of the external factors that affect the metabolic activity of microorganisms, it reduces the microbial action on *kocho* in KG kebele.

#### 3.2.2. Crude Fat

The analysis of variance shows that the fat content of the studied kocho samples is subject to all four factors, which is evident with the statistical significance (*p* ≤ 0.05) (Table 2). It shows that the model for the fat content of the study was statistically significant at a 100% confidence level and gives insight into the interaction of all the factors. In this study, adding gammicho reduced the crude fat content of kocho processed from both varieties in both Kebeles (Table 3). That is, except for the Meazia from DK remains, other samples (Meazia from KG Kebele, Katania from DK, and KG Kebeles) of their fat content of kocho treated with gammicho have significantly (*p* ≤ 0.05) higher than that of untreated kocho (shown in Table 4). The higher fat content in Meazia kocho (L_2_V_1_S_1_F_0_ has 1.69% fat) treated with gammicho before fermentation could be due to the fat content of gammicho together with the fat content of the scraped and pulverized enset mixture.

The crude fat content decreased significantly (*p* ≤ 0.05) with the elapsed fermentation time (after four months). As shown in Table 3, the crude fat content of Meazia and Katania *kocho* mixed with gammicho was reduced from 1.55 to 0.61% and from 1.26 to 0.31% as well as *kocho* without gammicho was also reduced from 1.57 to 0.68% and from 1.096 to 0.51% in Disa Kera, respectively. Similarly, the crude fat content of Meazia and Katania *kocho* mixed with gammicho was reduced from 1.69 to 0.80% and from 1.42 to 0.55% and *kocho* without gammicho in the two varieties was decreased from 1.26 to 0.77% and from 1.06 to 0.58% in Koysha Gorta Kebele. The reduction in the crude fat content of *kocho* during the fermentation process might be due to the utilization of fat by fermenting microbes as a source of energy. Furthermore, even after four months of fermentation, the crude fat content of the present study (ranging from 1.69 to 0.31%) was found as higher than the reported values of an earlier study [35]. This variation in the fat content of the present study might be attributed to the degree of fermentation, location, and variety of the crops.

#### 3.2.3. Crude Fiber Content

Crude fiber is the total amount of starchy polysaccharides such as cellulose, hemicellulose, lignin, and pectin substances that are not easily digested and absorbed in the body. The fiber content of the samples was analyzed through variance, which shows that the mean values differ significantly (*p* ≤ 0.001) in 3-factor interactions than in four-factor interactions (shown in Table 2). Furthermore, significant variations were evaluated to understand the effect of location, varieties, starter, and fermentation time by the response evaluation to fiber content.

The experimental results of ANOVA factorial analysis of fiber were analyzed and presented in Table 3. It indicates that the fiber content in fresh mass processed from the Meazia variety (L_1_V_1_F_0_: 9.74% and L_2_V_1_F_0_: 11.46%) was significantly higher than the Katania variety (L_1_V_2_F_0_: 7.46% and D_2_V_2_F_0_: 9.36%) in DK and KG Kebeles, respectively. This variation could be attributed to the genetic differences in which the enset of the Meazia variety might have higher cellulosic and lignin materials than the Katania variety. The results in the present study were consistent with the reported range, and the fiber content of enset varieties (red Bocho, white Bocho, and Epo) ranged from 5.46 to 21.48% [36].

Gammicho contributed its own effect (see Table 2) to the initial fiber content of unfermented mass. The experimental results confirmed that the initial amount of fiber in fresh Meazia and Katania pulps treated with gammicho (V_1_S_1_F_0_: 10.78% and (V_2_S_1_F_0_: 8.89%) was higher than that of the untreated mass of Meazia (V_1_S_2_F_0_: 10.42%) and Katania (V_2_S_2_F_0_: 7.92%), respectively. Similarly, gammicho-treated *kocho* samples showed a significant change in fiber content (shown in Table 3) compared with the untreated *kocho* studied in two locations (Disa Kera and Koysha Gorta). The higher fiber content of gammicho-treated fresh mass could be due to gammicho being the source of additional fiber.

The fiber content of *kocho* was significantly (*p* < 0.05) affected by the fermentation time. As incubation time increased, the crude fiber in fermenting mass steadily decreased (shown in Table 3). For instance, in Meazia *kocho*, fiber content was reduced from 10.78% (V_1_S_1_F_0_) to 4.57% (V_1_S_1_F_4_) and in Katania from 8.89% (V_2_S_1_F_0_) to 3.76% (V_2_S_1_F_4_) after four months of fermentation with gammicho. Even if fermentation was late and gradual in untreated mass, a similar reduction was taken place by the enzymatic activity of microbes developed in the fermentation products. That is, the crude fiber content of *kocho* fermented from Meazia local variety decreased from 9.74% (L_1_V_1_F_0_) to 5.56% (L_1_V_1_F_4_). Katania variety was from 7.46% (L_1_V_2_F_0_) to 5.17% (L_1_V_2_F_4_) in DK, whereas in Koysha Gorta, the fiber content of Meazia *kocho* was decreased from 11.46% (L_2_V_1_F_0_) to 5.03% (L_2_V_1_F_4_) and that of Katania *kocho* slightly increased from 3.08% (L_2_V_2_F_0_) to 4.77% (L_2_V_2_F_4_). This random variation could result from treatment with gammicho, which accelerated the partial degradation of cellulose, hemicelluloses, and lignin of *kocho* during fermentation periods because of the higher microbial load. The current result matches the earlier study by Alemu [37] in which the fiber content was reduced from 7.92 to 5.56%. Still, the variation in fiber content of the present study before and after fermentation might be due to the difference in variety, location, and length of fermentation.

These results were less than the upper limit of the average daily allowable dietary fiber for all ages recommended by the U.S. National Academy of Science report [38]. The highest fiber (D_2_V_1_F_0_: 11.46%) contained in a fresh mass of Meazia variety in Koysha Gorta, and the lowest fiber (D_1_V_2_F_4_: 2.85%) included in the fermented *kocho* of Katania variety in DK might be due to the difference in environmental conditions in the two Kebeles.

#### 3.2.4. Available Carbohydrate Content

In developing countries, carbohydrate is the body's primary energy source, primarily from plant products. Its amount varies with major food constituents, variety, and maturation stages. In traditionally fermented enset products (*kocho*), the available carbohydrate content varies with variety, gammicho, and fermentation time (Table 3).

Results shown in Table 3 reveal the variation in the amount of available carbohydrate of Katania (L_1_V_2_S_2_: 82.47% and L_2_V_2_S_2_: 80.05%) and Meazia varieties (L_1_V_1_S_2_: 79.75% and L_2_V_1_S_2_: 75.55%) is insignificant in DK and GK Kebeles, respectively. Some variations were noticed in carbohydrates that could be attributed to genetic differences that tissues of the Katania variety could store relatively higher starch with less water than Meazia. The available carbohydrate content of *kocho* in this study was higher than the values of the Astare variety (32.75%) and Kinnare variety (35.53%) as reported by others [36]. The difference could be due to variations in genetic composition, maturity, and place of cultivation. It was also higher than the dietary reference intake (DRI) values for carbohydrates in all ages (45–65%) [38].

The amount of total carbohydrate in fresh mass (scraped pulp) of Katania variety treated with gammicho is statistically lower (L_1_V_2_S_1_: 81.18% and L_2_V_2_S_1_: 78.38%) than untreated mass (L_1_V_2_S_2_: 82.47% and L_2_V_2_S_2_: 80.05%). Similarly, the fresh product processed from the Meazia variety with gammicho (L_1_V_1_S_1_: 77.97% and L_2_V_1_S_1_: 74.95%) has significantly (*p* < 0.05) lower carbohydrate percent than untreated content (L_1_V_1_S_2_: 79.75% and L_2_V_1_S_2_: 75.55%) in DK and GK Kebeles, respectively, due to higher activity, which may be utilized by the gammicho during the fermentation. Results reported in this study were slightly lower than earlier research done by Meseret [36], which was 84% for *kocho* flour but greater than the value for 72 hours of fermented maize dough (60.1-60.2%) [17].

The total carbohydrate amount in *kocho* fermented in KG was higher than that in Disa Kera. This might be attributed to the low and slow degradation [39] of carbohydrates in cold temperatures, as it is one of the growth factors for microbial cells. The highest value (82.24%) of carbohydrate in a fresh mass of Katania enset variety is processed from treated without gammicho in DK. The lowest value (78.69%) was obtained from four months of fermented *kocho* of the Meazia variety in Disa Kera. This might be due to low initial moisture and high dry mass content in fresh and fermented *kocho*.

#### 3.2.5. Energy Value

Gross energy is the total chemical energy stored in the chemical bonds of food released when the bonds are broken and used as fuel for cellular activities [40]. Statistically, Table 4 shows the 4-factorial interaction mean and the influences of all the four factors, such as location, variety, starter, and fermentation time. The results indicated that the gross energy (350.33 kcal/100 g in DK and 348.29 kcal/100 g in KG) of the scraped pulp of the Katania local variety was slightly higher than that of the Meazia variety.

Treatment with a starter culture (gammicho) influenced a significant change in the total energy density of fresh samples from 347.09 (L_2_V_2_S_2_F_0_) to 324.04 kcal (L_2_V_2_S_1_F_0_) in Katania and from 316.85 (L_2_V_1_S_2_F_0_) to 314.73 kcal (L_2_V_1_S_1_F_0_) in Meazia that is collected from the Koysha Gorta Kebele. Similarly, in Disa Kera, the total energy density of fresh masses was slightly reduced significantly from L_1_V_1_S_2_F_0_: 335.11 to L_1_V_1_S_1_F_0_: 323.45 kcal (in Meazia) and from L_2_V_2_S_2_F_0_: 347.09 to L_2_V_2_S_1_F_0_: 324.04 kcal/100 g (in Katania). The higher energy content of fresh mass with gammicho could be due to gammicho itself having an additional energy source.

The energy content of the studied *kocho* samples increased upon increasing the fermentation time. Even though the degradation of carbohydrates, fat, and fibers (energy sources of life) was decreased by microbial metabolism, the primary energy source of protein increased during the fermentation of *kocho*. Thus, the total energy contained in the untreated pulp of scraped and pulverized mixture of Meazia and Katania varieties in the Disa Kera location gets increased (see Table 4) from 335.11 (L_1_V_1_S_2_F_0_) to 354.86 kcal (L_1_V_1_S_2_F_4_) and from 331.35 (L_1_V_2_S_2_F_0_) to 364.47 kcal (L_1_V_2_S_2_F_4_) per 100 g dry mass of *kocho*. Also, the energy content of treated *kocho* gets improved significantly (shown in Table 4) from 323.45 (L_1_V_1_S_1_F_0_) to 352.83 kcal (L_1_V_1_S_1_F_4_) in Meazia and from 337.37 (L_1_V_2_S_1_F_0_) to 360.64 kcal/100 g ((L_1_V_2_S_1_F_4_) in Katania that is collected from DK Kebele. Similarly, the total energy contained in the untreated and treated pulp of scraped and pulverized mixture Meazia and Katania *kocho* samples had increased (shown in Table 4). The results of the present study were found as comparable with FAO/WHO, 2003 standard [17], and but it is less than the reported [6] in the literature, which might be due to the differences in genetic makeup, length of fermentation, and moisture content of the studied samples.

Since fermentation was slower in the highland (Koysha Gorta), high energy was stored compared to energy obtained by consuming a similar amount of *kocho* in the mid-highland (Disa Kera) due to the higher amounts of carbohydrate, protein, and fat. The highest gross energy (364.47 kcal/100 g) was obtained from four months of fermented Katania variety treated without gammicho in DK, while the most negligible value (314.73 kcal/100 g) was obtained from fresh unfermented *kocho* of Meazia variety in KG Kebele due to the considerable amount of carbohydrate (50-80%), fat, and protein contents that contribute a more significant energy share in the case of enset foods especially in *kocho* and bulla, whereas WHO/FAO recommended carbohydrate energy ratio for many foods as in the range of 50-75% [17].

### 3.3. Physicochemical Properties of Kocho

#### 3.3.1. Moisture Content

Moisture is an essential attribute in food quality and preservation. It is used to determine the percentage of other food constituents, such as total carbohydrates, on a wet and dry basis [39]. The results shown in Table 4 indicated that the moisture content of *kocho* samples of the two varieties is significantly affected by the varietal difference, gammicho catalyst, location temperature, and fermentation time. The moisture content (on a wet basis) of raw mass (scraped pulp) processed from the Meazia variety ranged from 75.06 to 78.28% and was significantly (*p* < 0.05) higher than the product of the Katania variety (70.34-75.47%) in DK and GK Kebeles, respectively. The observed variation in the initial moisture of the two varieties in two Kebeles could be the result of genetic variation, the water content of gammicho, and the relative humidity of the surrounding environment. As genetic variation is concerned, tissues and cells of the Meazia variety can hold more free and bound water than the Katania variety for cellular metabolism [40].

Enset plants have large mesophyll air/water spaces uniformly arranged between upper and lower fibrous spaces. As a result, the Meazia variety might have larger mesophyll air/water spaces and consequently contains higher moisture than Katania with smaller mesophyll air/water spaces between upper and lower fibrous spaces. In addition, enset plants have pockets in between each successive leaf sheath with different sizes depending on the size of the plant to hold water to overcome the challenges of dry seasons. The Meazia variety could have a large amount of water due to its large-sized pocket, which could also affect the moisture content of raw Meazia *kocho*. The soil moisture from adding animal dung and other household organic wastes into the enset garden was another factor to increase the water content in enset plants, especially Meazia local varieties, due to its high root density and surface area can harvest large amounts of water. The result of the present study was less than the moisture content of fresh *kocho* (85%) reported, which could be due to the difference in genetic composition [33, 41, 42], for instance, reported 84.4% of moisture on fresh *kocho*.

The addition of the gammicho starter has contributed to the moisture content (evaluated on a dry basis, shown in Table 4) of two varieties in two Kebeles. The water content of scraped pulp mixed with gammicho starter of Meazia variety (L_1_V_1_S_1_F_0_: 8.72% and L_2_V_1_S_1_F_0_: 9.34%) and Katania variety (L_1_V_2_S_1_F_0_: 7.32 and L_2_V_2_S_1_F_0_: 8.41%) was significantly (*p* < 0.05) higher than *kocho* processed without gammicho in Meazia (L_1_V_1_S_2_F_0_: 6.31% and L_2_V_1_S_2_F_0_: 8.62%) and Katania (L_1_V_2_S_2_F_0_: 5.44% and L_2_V_2_S_1_F_0_: 7.49%) varieties in DK and KG, respectively. This variation could be due to the water content of gammicho and the moisture content of the *kocho* varieties of samples.

Furthermore, the moisture content of *kocho* was significantly (*p* < 0.05) affected by the length of fermentation (shown in Table 4). The result confirmed that the percentage of moisture in *kocho* in all cases was reduced upon elevated fermentation time. Moisture content (on a wet and dry basis) of fresh mass with gammicho was significantly higher than fermented *kocho* in both Kebeles. The water content of fresh mass prepared from the Meazia variety ranged from 75.06 to 78.28% (on a wet basis) and as 8.72-9.34% (on a dry basis), and that of Katania is from 70.34 to 75.47% (in wet basis) and 5.44 to 8.41 (in dry basis). In contrast, the moisture content of four-month fermented *kocho* of Meazia variety ranged from 50.57 to 56.17% (on a wet basis) and 5.63 to 5.34% (on a dry basis) and Katania from 49.62 to 51.88% (in wet basis) and 4.68 to 4.74 (dry basis) in DK and KG, respectively. In the same way, the higher moisture contained in Meazia *kocho* fermented without gammicho and the lower amount of water was retained after four months of fermented *kocho* of Meazia variety and Katania in two Kebeles (shown in Table 4). It might be because the fermentation process started immediately (earlier) in *kocho* treated with gammicho, and the high density of fermenting microorganisms utilized much water for metabolic activities.

#### 3.3.2. The pH Value

According to FDA, 2013, acidic foods are foods that have natural pH of 4.6 and below. *Kocho*, ready for consumption, has an average pH value of less than 4.0. Hence, it is an acidic food with a sour taste compared to other traditional fermented foods of the microbial conversion of starch into organic acid. Still, the acidity level varies with variety, microbial load, and length of fermentation. According to the earlier study by Hiwot [43], in addition to organic acids developed through fermentation processes, the enset plant naturally contains weak organic acids, which improve the shelf life of processed *kocho* foods. The results in Table 4 showed that the acidity level of *kocho* varied with variety, fermentation time, and gammicho content.

As variety is concerned, the Meazia variety was more acidic (pH: 4.9 and 4.7) than the Katania variety (pH: 6.5 and 4.8) in DK and GK (Table 3), respectively. This might be attributed to the genetic differences that the Meazia variety contained more natural organic acids than the Katania variety. Treatment with gammicho increased the density of fermenting microorganisms, reducing pH, and increasing the acidity of studied foodstuffs (*kocho*). Thus, *kocho* of both varieties fermented with gammicho has a lowering pH value placed in a range of 4.5 (L_1_V_1_S_1_F_0_)-4.15 (L_2_V_1_S_1_F_0_) and 5.4 (L_1_V_2_S_1_F_0_)-4.51 (L_2_V_2_S_1_F_0_) than *kocho* fermented without gammicho, L_1_V_1_S_2_F_0_: 4.9-L_2_V_1_S_2_F_0_: 4.7 and L_1_V_2_S_2_F_0_: 6.5-L_2_V_2_S_2_F_0_: 4.8 in two Kebeles. It might be due to the higher fermenting microbes, mainly lactic acid bacteria (LAB). As indicated in the previous section, the *kocho* fermented with gammicho had a higher microbial load (microorganisms created from prefermented gammicho and developed during the fermentation process) that produced organic acids such as lactic acid, tartaric acid, acetic acid, propionic acid, and butyric acid, as by-products when microorganisms ferment carbohydrates for growth and multiplication. Similar results were reported [42] that the pH value of raw *kocho* treated with gammicho was lower than that fermented without the starter culture.

As fermentation time increases, the pH value of *kocho* decreases (shown in Table 4). That is, the pH value of *kocho* processed from Meazia and Katania varieties without gammicho catalyst was reduced from 4.9 to 3.33 in DK Kebele and from 4.7 to 3.48 in KG Kebele and 6.5 to 3.23 (in DK) and from 4.8 to 2.99 (in KG) after four months of fermentation. This might be attributed to the accumulation of organic acids, mainly lactic and acetic acids, as *kocho* is subjected to fermenting microorganisms. This result agreed with a slight deviation from the results reported by Ashenafi [44]. Moreover, gammicho addition, along with long-time fermentation, caused much reduction in the pH of fermented mass, which is prone in their results during the four months of fermentation in both Kebeles (Table 4).

#### 3.3.3. Titrable Acidity

Titrable acidity (TA) is the percentage of lactic acid present in fermented mass and has an inverse relation with pH value. Variety, crop cultivation location, fermentation time, and gammicho affect the acidity level of *kocho*. The total acid concentration in the Meazia variety was slightly more significant than that of the Katania variety in both Kebeles (Table 3). It is because of the genetic variation that Meazia is naturally more acidic than Katania. The TA value of Meazia *kocho* (0.13%) is greater than Katania *kocho* (0.09%) in DK and KG Kebeles, respectively.

The amount of titrable acidity in the present study of *kocho* fermented for four months with gammicho was increased (Table 3) in many folds (i.e., TA value of Meazia *kocho* in DK was increased from 0.13 (V_1_F_0_) to 0.27% (V_1_F_2_) in two months of fermentation and further increased to 0.41% (V_1_F_4_) after four months of fermentation). Similarly, in Katania *kocho*, from 0.09 (V_2_F_0_) to 0.23% (V_2_F_2_) in two months of fermentation, it further increased to 0.37% (V_2_F_4_) after four months. It could be due to the accumulation of organic acids, mainly lactic acid, produced by lactic acid bacteria, as the primary end product of carbohydrate fermentation in *kocho*. A similar trend with a gradual increment in titrable acidity was observed in both Meazia and Katania *kocho* fermented without gammicho in KG Kebele. Such a trend was also reported with the fermentation of teff and wheat [45]. The gradual increase in acidity of *kocho* in KG Kebele might be due to the low microbial load and its slow activity due to cold temperatures. Higher TA in *kocho* processed and fermented in DK was due to higher microbial load and their fast action due to suitable environmental conditions for the growth and multiplication of fermenting microorganisms. The enhancement of acidity in *kocho* was mainly due to the conversion of sugar into alcohol and organic acids as by-products of LAB and some species of yeast and molds.

#### 3.3.4. Ash Content

According to the ASEAN Manual of Food Analysis [46], the ash content of a given substance is the percent of inorganic residue left after incineration of organic matter and use parameters in the nutritional value of given foods. The results (Table 4) indicated that the percent of ash in raw Meazia pulp (ash: 2.35 g and 2.44 g) is higher than that of raw Katania pulp (ash: 2.07 g and 2.24 g) in DK and KG Kebeles, respectively. It could be due to the absorbing minerals through roots and storing nutrients. The Meazia variety has fibrous roots with dense root hairs and large surface areas to absorb minerals from the soil for different life activities. It has many storage sites that store these minerals.

As the fermentation time increase, the ash content of *kocho* also increases. The result showed (Table 4) that the ash content of *kocho* of Meazia variety treated with gammicho rose from 2.41 g to 3.57 g and 2.46 g to 3.49 g in DK and KG Kebeles, respectively. Similarly, the ash content of Katania *kocho* increased from 2.07 g to 3.33 g in DK and from 2.41 g to 3.21 g in KG Kebeles after four months of fermentation. On the other hand, the ash content of untreated *kocho* of the two varieties was also increased similarly. An increase in crude ash could be due to the removal of antinutritional factors and the dissociation of mineral-nutrient complexes during the fermentation stages. Moreover, the increment might be related to the reduction in moisture content and an increment of dry matter. The trend also agrees with related reports [17, 43].

## 4. Conclusions

The outcome showed that the Meazia variety has higher crude protein, fat, fiber, moisture, pH, TA, and ash levels. In comparison, the Katania type in both Kebeles has a high level of gross calories and total carbohydrates. In comparison to the prior stated literature values, they have increased nutritious values. The fermentation period has an impact on both varieties of kocho's physicochemical and nutritional characteristics. The moisture content, pH level, crude fat, crude fiber, total carbohydrate, and calorie values of kocho dropped as fermentation time rose, with the exception of the crude protein and ash. Thus, kocho that has been fermented for four months (120 days) has more nutritious value than kocho that has only been fermented for two months. The findings show that gammicho has a marginally significant impact on nutritional content and physicochemical qualities, in addition to promoting fermentation by increasing the kocho's microbial population and protein content. Consequently, gammicho treatment during the fourth month of fermentation is preferable to the second month for generating high-quality kocho.

## Figures and Tables

**Table 1:** Phytochemical screening of the Meazia and Katania varieties of two different locations.

Active compounds	Sample location and varieties of enset			
	Disa Kera		Koysha Gorta	
	Meazia	Katania	Meazia	Katania
Tannins	+++	++	+++	++
Saponins	+++	+++	+++	++
Alkaloids	++	+	++	+
Phenols	++	++	++	++
Terpenoids	+	-	+	-
Flavonoids	+	+	+	+
Volatile oils	-	-	-	-

Note: +++: high amount, shown immediately after added reagent; ++: moderate amount after 5 minutes; +: low amount after 10 minutes of reagent added; -: absent of active compound.

**Table 2:** ANOVA results (*p* values) for the proximate and physicochemical composition of *kocho.*

Source of factors	Protein	Fat	Fiber	Moisture	Ash	CHO	Energy	pH	TA
Loc	0.378	≤0.001	≤0.001	≤0.001	≤0.001	≤0.001	≤0.001	≤0.001	≤0.001
Var	≤0.001	≤0.001	≤0.001	≤0.001	≤0.001	≤0.001	≤0.001	≤0.001	0.088
Strt	≤0.001	≤0.001	≤0.001	≤0.001	≤0.001	≤0.001	≤0.001	≤0.001	0.001
FT	≤0.001	≤0.001	≤0.001	≤0.001	≤0.001	≤0.001	≤0.001	≤0.001	0.000
Loc^∗^Var	0.412	≤0.001	≤0.001	0.138	≤0.001	≤0.001	≤0.001	≤0.001	0.055
Loc^∗^Strt	0.067	≤0.001	0.362	≤0.001	0.840	0.041	≤0.001	≤0.001	0.017
Loc^∗^FT	≤0.001	≤0.001	≤0.001	≤0.001	≤0.001	≤0.001	≤0.001	≤0.001	0.015
Var^∗^Strt	0.001	0.369	0.096	≤0.001	0.545	0.151	≤0.001	0.194	0.133
Var^∗^FT	0.678	0.022	≤0.001	≤0.001	≤0.001	≤0.001	≤0.001	≤0.001	0.039
Strt^∗^FT	≤0.001	≤0.001	≤0.001	≤0.001	≤0.001	≤0.001	≤0.001	≤0.001	0.006
Loc^∗^Var^∗^Strt	0.752	0.003	0.026	0.001	≤0.001	≤0.001	≤0.001	≤0.001	0.614
Loc^∗^Var^∗^FT	≤0.001	0.002	≤0.001	0.165	≤0.001	≤0.001	≤0.001	≤0.001	0.153
Loc^∗^Strt^∗^FT	0.364	≤0.001	≤0.001	0.003	0.013	0.673	≤0.001	≤0.001	0.729
Var^∗^Strt^∗^FT	0.489	0.031	≤0.001	≤0.001	≤0.001	0.088	≤0.001	≤0.001	0.134
Loc^∗^Var^∗^Strt^∗^FT	0.453	≤0.001	0.407	0.011	≤0.001	0.516	0.004	≤0.001	0.062

*R*-squared (%)	98.07	98.84	99.32	99.02	99.63	99.21	99.74	99.90	81.46
*R*-squared adj. (%)	97.14	98.28	99.00	98.55	99.45	98.81	99.61	99.86	72.58

Note: Loc: location; Var: varieties of *kocho*; Strt: starter culture; FT: fermentation time; CHO: carbohydrates; TA: titrable acidity.

**(a):** 

Source of two-factorial interactions:											
Loc^∗^Strt	TA	Loc^∗^FT	TA	CHO	Var^∗^Strt	Protein	Var^∗^FT	TA	Strt^∗^FT	TA	Protein
L_1_S_1_	0.38^a^	L_1_F_4_	0.47^a^	82.99^a^	V_1_S_1_	4.49^a^	V_1_F_4_	0.41^a^	S_1_F_4_	0.45^a^	5.52^a^
L_2_S_1_	0.22^b^	L_2_F_4_	0.31^bc^	80.51^b^	V_2_S_1_	4.01^b^	V_2_F_4_	0.37^a^	S_2_F_4_	0.33^b^	4.75^b^
L_1_S_2_	0.25^b^	L_2_F_2_	0.21^cd^	77.21^c^	V_1_S_2_	3.96^b^	V_1_F_2_	0.33^ab^	S_1_F_2_	0.35^ab^	3.82^c^
L_2_S_2_	0.19^b^	L_1_F_2_	0.35^b^	80.35^b^	V_2_S_2_	3.72^c^	V_2_F_2_	0.23^bc^	S_2_F_2_	0.21^c^	3.59^d^
		L_2_F_0_	0.11^d^	73.96^d^			V_1_F_0_	0.09^d^	S_1_F_0_	0.10^c^	3.42^d^
		L_1_F_0_	0.12^d^	77.68^c^			V_2_F_0_	0.13^cd^	S_2_F_0_	0.12^c^	3.19^e^

**(b):** 

Source of three-factorial interactions:										
Loc^∗^Var^∗^FT	Protein	Fiber	CHO	Loc^∗^Var^∗^Strt	Fiber	CHO	Loc^∗^Strt^∗^FT	Fiber	Var^∗^Strt^∗^FT	Fiber
L_1_V_1_F_4_	5.56^a^	3.74^h^	81.44^b^	L_2_V_1_S_1_	8.76^a^	74.95^f^	L_1_S_1_F_4_	2.79^i^	V_1_S_1_F_4_	4.57^g^
L_1_V_2_F_4_	5.17^b^	2.85^i^	84.55^a^	L_1_V_1_S_1_	6.43^d^	77.97^d^	L_2_S_1_F_4_	5.53^g^	V_2_S_1_F_4_	3.76^h^
L_2_V_1_F_4_	5.03^bc^	5.79^fg^	79.06^c^	L_2_V_2_S_1_	7.01^bc^	78.38^d^	L_1_S_2_F_4_	3.79^h^	V_1_S_2_F_4_	4.96^g^
L_2_V_2_F_4_	4.77^c^	5.50^g^	81.97^b^	L_1_V_2_S_1_	5.28^f^	81.18^b^	L_2_S_2_F_4_	5.762^g^	V_2_S_2_F_4_	4.59^g^
L_2_V_1_F_2_	3.90^d^	9.58^b^	74.81^e^	L_1_V_1_S_2_	6.64^cd^	79.75^c^	L_2_S_1_F_2_	7.33^e^	V_1_S_1_F_2_	7.42^de^
L_1_V_1_F_2_	3.88^d^	6.12^efg^	79.59^c^	L_2_V_1_S_2_	9.13^a^	75.55^e^	L_1_S_1_F_2_	5.88^g^	V_1_S_2_F_2_	8.28^c^
L_2_V_1_F_0_	3.75^d^	11.46^a^	71.87^f^	L_1_V_2_S_2_	5.97^e^	82.47^a^	L_2_S_2_F_2_	8.88^c^	V_1_S_1_F_0_	10.78^a^
L_2_V_2_F_2_	3.65^de^	6.63^d^	79.62^c^	L_2_V_2_S_2_	7.31^b^	80.05^c^	L_2_S_1_F_0_	10.79^a^	V_2_S_1_F_2_	5.78^f^
L_1_V_2_F_2_	3.41^ef^	6.57^de^	81.12^b^				L_1_S_2_F_2_	6.81^f^	V_2_S_2_F_2_	7.42^e^
L_1_V_1_F_0_	3.26^fg^	9.74^b^	75.56^de^				L_1_S_1_F_0_	8.89^c^	V_1_S_2_F_0_	10.42^a^
L_1_V_2_F_0_	3.12^fg^	7.46^c^	79.79^c^				L_2_S_2_F_0_	10.03^b^	V_2_S_1_F_0_	8.89^b^
L_2_V_2_F_0_	3.08^g^	9.36^b^	76.05^d^				L_1_S_2_F_0_	8.30^d^	V_2_S_2_F_0_	7.92^cd^

Note: L_1_, L_2_: location: L_1_: Disa Kera; L_2_: Koysha Gorta; V_1_, V_2_: *kocho* variety: V_1_: Meazia; V_2_: Katania; S_1_, S_2_: starter culture: S_1_: with gamma; S_2_: without gamma; F_0_, F_2_, and F_4_: fermentation time: F_0_: nonfermented *kocho* (initial); F_2_: 2-month fermented *kocho*; F_4_: 4-month fermented *kocho*. Means within the column with different letters are significantly different (*p* < 0.05).

**Table 4:** Proximate composition and physical characteristics of traditionally fermented *kocho* Ethiopian false banana foodstuff (four-factorial interactions).

Loc^∗^Var^∗^Strt^∗^FT	Mean values (%)					
	Fat	Moisture (wet basis)	Moisture (dry basis)	Energy	pH	Ash
L_1_V_1_S_1_F_4_	0.62^hij^	50.57^o^	5.63^f^	352.83^c^	3.00^p^	3.57^a^
L_2_V_1_S_1_F_4_	0.80^g^	56.17^l^	5.34^fg^	345.66^efg^	3.20^o^	3.49^ab^
L_1_V_2_S_1_F_4_	0.31^k^	49.62^o^	4.68^h^	360.64^b^	3.20^o^	3.33^cd^
L_1_V_1_S_2_F_4_	0.68^ghi^	55.06^m^	4.69^h^	354.86^c^	3.54^lm^	3.33^cd^
L_2_V_2_S_1_F_4_	0.55^ij^	51.88^n^	4.74^gh^	349.75^d^	3.50^m^	3.21^e^
L_1_V_2_S_2_F_4_	0.51^j^	52.04^n^	2.81^j^	364.47^a^	3.60^l^	3.23^de^
L_2_V_1_S_2_F_4_	0.77^gh^	58.09^k^	6.37^de^	316.85^n^	3.40^n^	3.48^ab^
L_2_V_2_S_2_F_4_	0.58^ij^	54.96^m^	3.45^i^	354.33^c^	3.60^l^	2.99^g^
L_1_V_1_S_1_F_2_	1.20^de^	60.16^j^	6.54^d^	342.83^h^	3.80^k^	3.18^ef^
L_2_V_1_S_1_F_2_	1.35^cd^	64.76^i^	7.48^c^	327.75^l^	3.61^l^	3.39^bc^
L_2_V_1_S_1_F_0_	1.69^a^	78.28^a^	9.34^a^	314.73^n^	4.15^h^	2.46^j^
L_2_V_1_S_2_F_2_	0.97^fg^	68.95^f^	7.29^c^	322.79^m^	3.90^j^	2.94^gh^
L_2_V_2_S_1_F_2_	1.32^cd^	67.02^h^	6.61^d^	344.65^efg^	3.80^k^	3.10^f^
L_1_V_1_S_2_F_2_	1.32^cd^	68.12^fg^	5.80^ef^	347.12^de^	4.21^h^	2.80^i^
L_2_V_2_S_2_F_2_	0.65^hij^	67.72^gh^	5.66^f^	339.28^hi^	4.00^i^	2.85^hi^
L_2_V_1_S_2_F_0_	1.26^d^	74.32^c^	8.62^b^	316.85^n^	4.70^e^	2.44^jk^
L_1_V_2_S_1_F_2_	0.62^hij^	58.22^k^	5.87^ef^	343.92^fg^	3.90^j^	3.14^ef^
L_1_V_1_S_1_F_0_	1.55^ab^	75.06^bc^	8.72^ab^	323.45^m^	4.50^f^	2.41^jk^
L_1_V_2_S_2_F_2_	1.08^ef^	65.48^i^	4.32^h^	347.60^def^	4.31^g^	2.78^i^
L_2_V_2_S_1_F_0_	1.42^bc^	75.47^b^	8.41^b^	324.04^m^	4.51^f^	2.41^jk^
L_1_V_2_S_1_F_0_	1.26^d^	70.34^e^	7.32^c^	337.37^ij^	5.40^b^	2.07^m^
L_1_V_1_S_2_F_0_	1.57^ab^	72.72^d^	6.31^de^	335.11^j^	4.90^c^	2.35^k^
L_1_V_2_S_2_F_0_	1.09^ef^	69.04^f^	5.44^f^	331.35^k^	6.50^a^	2.07^m^
L_2_V_2_S_2_F_0_	1.06^ef^	72.36^d^	7.49^c^	347.09^de^	4.80^d^	2.24^l^

Note: L_1_, L_2_: location: L_1_: Disa Kera; L_2_: Koysha Gorta; V_1_, V_2_: *kocho* variety: V_1_: Meazia; V_2_: Katania; S_1_, S_2_: starter culture: S_1_: with gamma; S_2_: without gamma; F_0_, F_2_, and F_4_: fermentation time: F_0_: unfermented *kocho* (initial); F_2_: 2 months fermented *kocho*; F_4_: 4 months fermented *kocho*. Means within the column with different letters are significantly different (*p* < 0.05).

## Data Availability

All the data have been included in the manuscript, and crude data were separately given as a data set. The table given as a supplementary data set includes the triplicate measurement of the physicochemical and nutraceutical values of the analyzed samples.

## Supplementary Materials

Supplementary Materials All the data have been included in the manuscript, and crude data were separately given as a data set. The table given as a supplementary data set includes the triplicate measurement of physicochemical and nutraceutical values of the analyzed samples.
