# Supplementary material for: Phytochemical Screening and Multivariate Analysis on Physicochemical and Nutraceutical Value of Kocho from False Banana (Enset)
Source: Int J Food Sci. 2023 Mar 9;2023:6666635. doi: 10.1155/2023/6666635 (PMC10019968; doi:10.1155/2023/6666635)
Supplement: Supplementary Materials — All the data have been included in the manuscript, and crude data were separately given as a data set. The table given as a supplementary data set includes the triplicate measurement of physicochemical and nutraceutical values of the analyzed samples. [file 6666635.f1.pdf]

Data set Table: The triplicate measurement of physicochemical and nutraceutical values of analyzed samples.

S/n	Location	Var	strt	FT	Triplicate	Protein	Fat	Fiber	Mc (db)	CHO	Energy	pH	TA	Ash
1	L1	V1	S1	F0	1	3.36	1.49	9.93	8.835	74.005	322.87	4.5	0.13	2.38
2	L1	V1	S1	F0	2	3.38	1.55	9.94	8.656	74.064	323.726	4.5	0.13	2.41
3	L1	V1	S1	F0	3	3.4	1.61	9.95	8.682	73.918	323.762	4.5	0.13	2.44
4	L1	V1	S1	F2	1	3.95	1.19	5.55	6.129	80.011	346.554	3.8	0.272	3.17
5	L1	V1	S1	F2	2	4.06	1.2	5.97	6.642	78.948	342.832	3.8	0.78	3.18
6	L1	V1	S1	F2	3	4.15	1.21	6.39	6.834	78.226	340.394	3.8	0.84	3.19
7	L1	V1	S1	F4	1	5.9	0.61	2.93	5.893	81.117	353.558	3	0.517	3.55
8	L1	V1	S1	F4	2	5.97	0.62	3.37	5.961	80.509	351.496	3	0.559	3.57
9	L1	V1	S1	F4	3	6.04	0.63	3.81	5.028	80.902	353.438	3	0.601	3.59
10	L1	V1	S2	F0	1	2.7	1.42	9.37	6.268	77.902	335.188	4.9	0.1129	2.34
11	L1	V1	S2	F0	2	3.32	1.57	9.53	6.352	76.878	334.922	4.9	0.1199	2.35
12	L1	V1	S2	F0	3	3.37	1.72	9.69	6.296	76.564	335.216	4.9	0.1269	2.36
13	L1	V1	S2	F2	1	3.63	1.31	6.18	5.834	80.266	347.374	4.2	0.256	2.78
14	L1	V1	S2	F2	2	3.7	1.32	6.28	5.627	80.273	347.772	4.21	0.271	2.8
15	L1	V1	S2	F2	3	3.77	1.33	6.36	5.931	79.789	346.206	4.22	0.286	2.82
16	L1	V1	S2	F4	1	4.87	0.67	4.04	4.726	82.374	355.006	3.44	0.402	3.32
17	L1	V1	S2	F4	2	5.15	0.68	4.11	4.528	82.202	355.528	3.54	0.408	3.33
18	L1	V1	S2	F4	3	5.43	0.69	4.18	4.83	81.53	354.05	3.64	0.414	3.34
19	L1	V2	S1	F0	1	3.04	1.24	7.67	7.228	78.762	338.368	5.3	0.121	2.06
20	L1	V2	S1	F0	2	3.14	1.26	7.84	7.351	78.339	337.256	5.4	0.124	2.07
21	L1	V2	S1	F0	3	3.24	1.28	8.01	7.392	77.998	336.472	5.5	0.127	2.08
22	L1	V2	S1	F2	1	3.32	0.59	5.53	5.94	81.49	344.55	3.9	0.26	3.13
23	L1	V2	S1	F2	2	3.46	0.62	5.78	5.826	81.174	344.116	3.9	0.27	3.14
24	L1	V2	S1	F2	3	3.6	0.65	6.03	5.859	80.711	343.094	3.9	0.28	3.15
25	L1	V2	S1	F4	1	5.39	0.28	2.21	4.862	83.938	359.832	3.2	0.447	3.32
26	L1	V2	S1	F4	2	5.41	0.31	2.22	4.381	84.349	361.826	3.2	0.562	3.33
27	L1	V2	S1	F4	3	5.43	0.34	2.23	4.793	83.867	360.248	3.2	0.677	3.34
28	L1	V2	S2	F0	1	3	1.07	7.06	5.262	81.568	347.902	6.5	0.104	2.04
29	L1	V2	S2	F0	2	3.09	1.09	7.08	5.688	80.982	346.098	6.5	0.113	2.07
30	L1	V2	S2	F0	3	3.18	1.11	7.1	5.372	81.138	347.262	6.5	0.122	2.1
31	L1	V2	S2	F2	1	3.18	0.96	6.43	4.238	82.432	351.088	4.3	0.23	2.76
32	L1	V2	S2	F2	2	3.35	1.08	7.35	4.319	81.121	347.604	4.31	0.24	2.78
33	L1	V2	S2	F2	3	3.52	1.2	8.27	4.397	79.813	344.132	4.32	0.25	2.8
34	L1	V2	S2	F4	1	4.8	0.49	3.46	2.985	85.045	363.79	3.6	0.34	3.22
35	L1	V2	S2	F4	2	4.92	0.51	3.48	2.824	85.036	364.414	3.6	0.34	3.23
36	L1	V2	S2	F4	3	5.04	0.53	3.5	2.625	85.065	365.19	3.6	0.34	3.24
37	L2	V1	S1	F0	1	3.84	1.69	11.63	9.165	71.225	315.47	4.14	0.08	2.45
38	L2	V1	S1	F0	2	4.01	1.69	11.63	9.537	70.673	313.942	4.15	0.08	2.46
39	L2	V1	S1	F0	3	4.18	1.69	11.63	9.317	70.713	314.782	4.16	0.08	2.47

NOTE:

Mc (db): Moisture content (dry basis)
CHO: Carbohydrates
TA: Titrable acidity

Location (place name):
L1 - Disa Kera
L2 - Koysha Gorta

Var (Variety of kocho sample):
V1 - Meazia
V2 - Katania

Strt. (Starter culture gamma):
S1 - with gamma
S2 - without gamma

FT (Fermentation time)
F0 - non-fermented kocho (initial)
F2 - 2 months fermented kocho
F4 - 4 months fermented kocho

40	L2	V1	S1	F2	1	3.88	1.34	8.68	7.382	75.338	328.932	3.6	0.25	3.38
41	L2	V1	S1	F2	2	4.01	1.35	8.88	7.53	74.84	327.55	3.61	0.26	3.39
42	L2	V1	S1	F2	3	4.14	1.36	9.08	7.526	74.494	326.776	3.62	0.27	3.4
43	L2	V1	S1	F4	1	5.47	0.74	5.61	5.387	79.323	345.832	3.2	0.33	3.47
44	L2	V1	S1	F4	2	5.54	0.8	5.76	5.138	79.272	346.448	3.2	0.35	3.49
45	L2	V1	S1	F4	3	5.61	0.86	5.91	5.48	78.63	344.7	3.2	0.37	3.51
46	L2	V1	S2	F0	1	3.36	1.21	11.29	8.356	73.354	317.746	4.68	0.06	2.43
47	L2	V1	S2	F0	2	3.5	1.26	11.3	8.821	72.679	316.056	4.7	0.06	2.44
48	L2	V1	S2	F0	3	3.64	1.31	11.31	8.691	72.599	316.746	4.72	0.06	2.45
49	L2	V1	S2	F2	1	3.61	0.93	10.2	7.244	75.096	323.194	3.9	0.162	2.92
50	L2	V1	S2	F2	2	3.79	0.97	10.28	7.183	74.837	323.238	3.9	0.168	2.94
51	L2	V1	S2	F2	3	3.97	1.01	10.36	7.457	74.243	321.942	3.9	0.174	2.96
52	L2	V1	S2	F4	1	4.49	0.74	5.79	6.47	79.05	340.82	3.4	0.3	3.46
53	L2	V1	S2	F4	2	4.52	0.77	5.82	6.13	79.28	342.13	3.4	0.32	3.48
54	L2	V1	S2	F4	3	4.55	0.8	5.85	6.518	78.782	340.528	3.4	0.34	3.5
55	L2	V2	S1	F0	1	2.99	1.42	9.77	8.26	75.16	325.38	4.5	0.08	2.4
56	L2	V2	S1	F0	2	3.15	1.42	9.95	8.417	74.653	323.992	4.51	0.08	2.41
57	L2	V2	S1	F0	3	3.31	1.42	10.13	8.538	74.182	322.748	4.52	0.08	2.42
58	L2	V2	S1	F2	1	3.7	1.27	5.53	6.772	79.458	344.062	3.8	0.24	3.27
59	L2	V2	S1	F2	2	3.77	1.32	5.78	6.524	79.606	345.384	3.8	0.25	3
60	L2	V2	S1	F2	3	3.84	1.37	6.03	6.53	79.2	344.49	3.8	0.26	3.03
61	L2	V2	S1	F4	1	5.01	0.53	5.27	4.849	81.161	349.454	3.5	0.3	3.18
62	L2	V2	S1	F4	2	5.14	0.55	5.3	4.792	81.008	349.542	3.5	0.31	3.21
63	L2	V2	S1	F4	3	5.27	0.57	5.33	4.582	81.008	350.242	3.5	0.32	3.24
64	L2	V2	S2	F0	1	2.79	0.99	8.72	7.518	77.752	331.078	4.8	0.043	2.23
65	L2	V2	S2	F0	2	3.02	1.06	8.76	7.628	77.292	330.788	4.8	0.048	2.24
66	L2	V2	S2	F0	3	3.25	1.13	8.8	7.32	77.25	332.17	4.8	0.53	2.25
67	L2	V2	S2	F2	1	3.52	0.62	7.46	5.841	79.729	338.576	4	0.15	2.83
68	L2	V2	S2	F2	2	3.54	0.65	7.48	5.619	79.861	339.454	4	0.16	2.85
69	L2	V2	S2	F2	3	3.56	0.68	7.5	5.527	79.863	339.812	4	0.17	2.87
70	L2	V2	S2	F4	1	4.33	0.56	5.51	3.286	83.344	355.736	3.6	0.26	2.97
71	L2	V2	S2	F4	2	4.39	0.58	5.77	3.489	82.781	353.904	3.6	0.26	2.99
72	L2	V2	S2	F4	3	4.45	0.6	5.83	3.575	82.535	353.34	3.6	0.26	3.01
